# Acute Respiratory Distress Syndrome Secondary to Miliary Tuberculosis

**DOI:** 10.4269/ajtmh.24-0808

**Published:** 2025-09-04

**Authors:** Kazuhisa Yokota

**Affiliations:** ^1^ Department of Infectious diseases, Tokyo Bay Urayasu Ichikawa Medical Center, Todaijima, Urayasu-city, Chiba, Japan;; ^2^ Department of Infectious diseases, Tokyo Metropolitan Ebara Hospital, Higashiyukigaya, Ota-ku, Tokyo, Japan

A 30-year-old woman with a late pregnancy was admitted with labor pain and a persistent fever for 2 weeks. Her medical history included treatment for latent tuberculosis infection five years prior. Upon admission, she underwent an emergency cesarean section due to obstetric indications.

**Figure 1.**
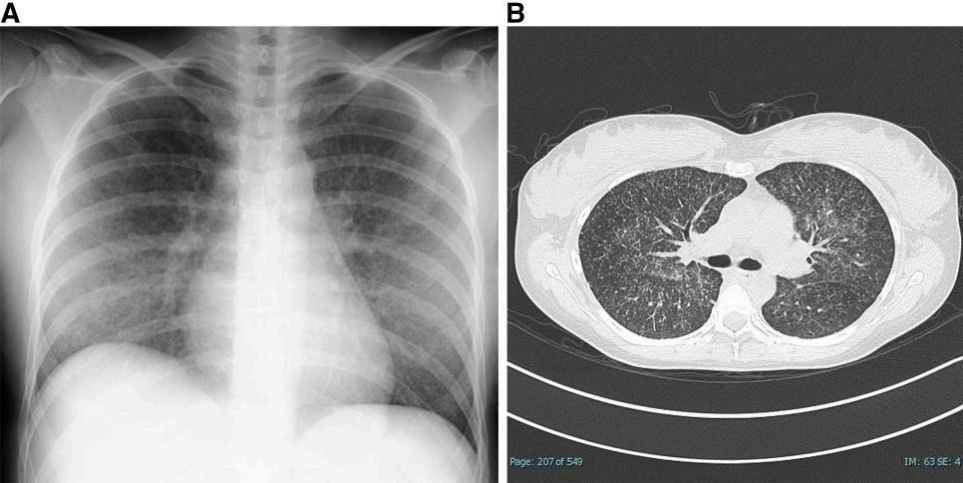
(**A**) CXR on day 2 showed patchy infiltrates and tiny diffuse nodules throughout the lungs consistent with miliary tuberculosis. (**B**) Chest CT scan on day 2. Diffuse small granular nodules are seen in the lung fields consistent with miliary tuberculosis.

On the second day of admission, a chest X-ray (CXR) and a computed tomography (CT) scan revealed small diffuse granular nodules throughout both lungs (Figure 1). Sputum and urine tests were positive for* Mycobacterium tuberculosis* (Mtb) on polymerase chain-reaction testing, and these cultures were also positive for Mtb although the acid-fast stain smears of her sputum presented negative for 3 consecutive days. A diagnosis of miliary tuberculosis was made. Antituberculosis therapy (rifampin, isoniazid, ethambutol, pyrazinamide, and levofloxacin) was initiated promptly. Given the failure of previous latent tuberculosis treatment and the possibility of drug resistance, levofloxacin was added to the standard antituberculosis treatment. It was later reported that the bacteria were susceptible to all of these drugs.

**Figure 2.**
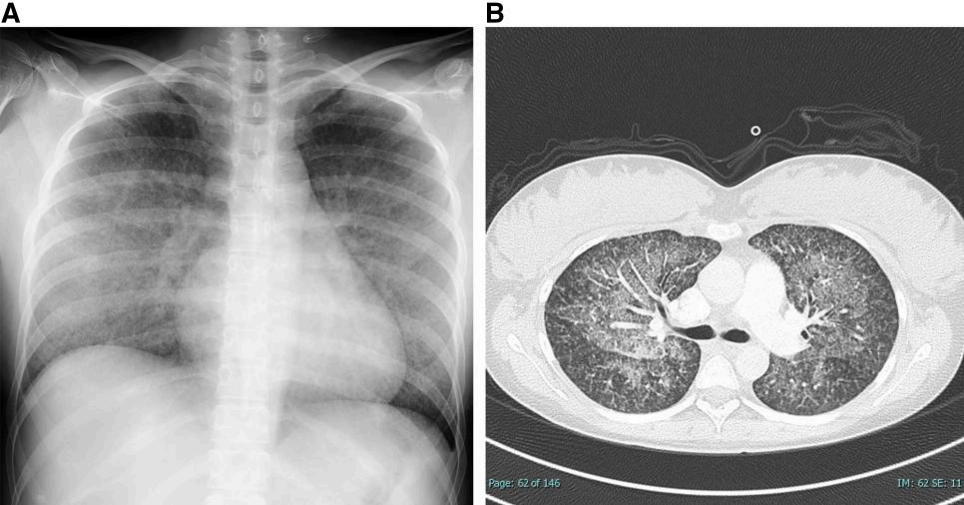
(**A**) CXR on day 6 (initiation day of steroid pulse therapy) revealed diffuse, homogenous, finely granular, cluster-like ground-glass opacities predominantly in the mid-to-lower lung fields. (**B**) CT scan on day 6. Extensive ground-glass opacities along with miliary shadows, consistent with ARDS secondary to miliary tuberculosis.

Two days after starting antituberculosis therapy, the patient experienced progressive respiratory distress, requiring an escalated treatment course of oxygen support. Oxygen supplementation reached 10 L/minute with a non-rebreather mask. A repeat CXR and a CT scan demonstrated widespread ground-glass opacities and tiny nodules (Figure 2), consistent with acute respiratory distress syndrome (ARDS) secondary to miliary tuberculosis.

**Figure 3.**
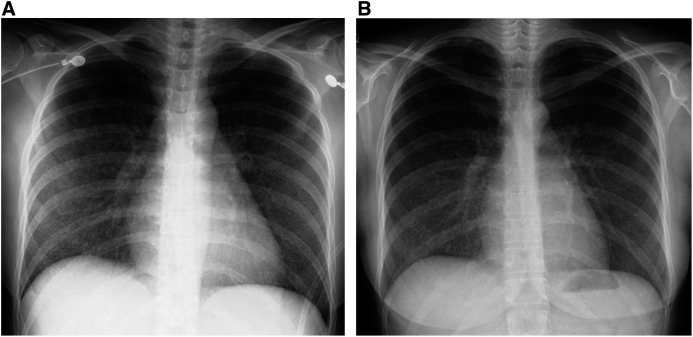
(**A**) CXR on day 9 (the day after completion of steroid pulse therapy). CXR showing marked improvement in bilateral pulmonary infiltrates following three days of steroid pulse therapy. (**B**) CXR on day 16 (on day 8 following the completion of steroid pulse therapy, the patient was undergoing steroid tapering). CXR showing marked improvement in bilateral pulmonary infiltrates during steroid tapering.

**Figure 4.**
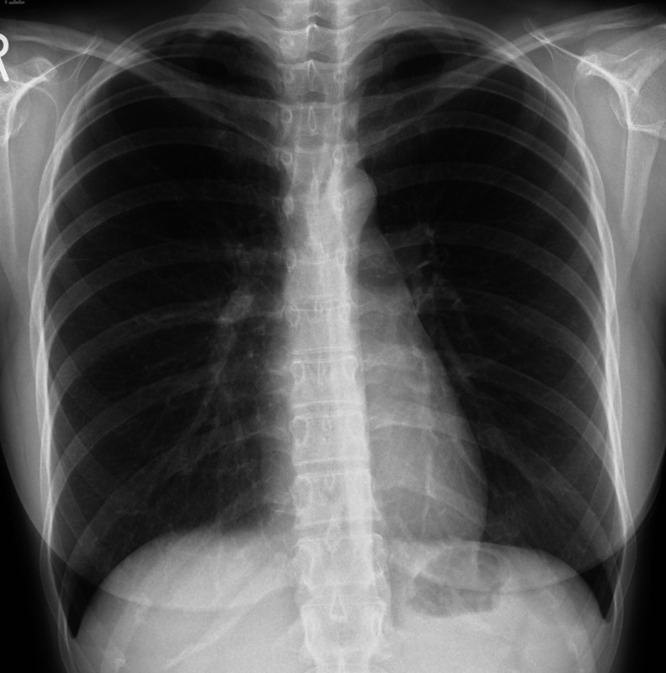
Follow-up CXR taken after completion of 6 months of antituberculosis therapy showing complete resolution of the previously seen miliary pattern.

Despite the administration of antituberculosis drugs for 2 days, her respiratory symptoms rapidly worsened, leading to ARDS. It was determined that antituberculosis drugs alone would not improve the situation, and the patient was treated with intravenous methylprednisolone for three days, followed by a tapering course of oral prednisolone. Her symptoms abated rapidly, with subsequent radiographic improvement observed on CXR (Figure 3). Two months after the initiation of antituberculosis treatment, the regimen was de-escalated to rifampin and isoniazid only and continued for another four months to complete a six-month course. Follow-up CXR after treatment ultimately confirmed resolution of the miliary pattern (Figure 4).

The incidence of ARDS secondary to miliary tuberculosis is reported to be 18.0%.^1^ Of these, 3.5%^1^ were pregnant women, making it a rare occurrence. However, when a pregnant woman presents with miliary tuberculosis, the possibility of ARDS complications should be carefully monitored. Whether or not to use steroid pulse therapy in these patients is also a matter of debate.^2^^,^^3^
